# Thermoelastic Processes by a Continuous Heat Source Line in an Infinite Solid via Moore–Gibson–Thompson Thermoelasticity

**DOI:** 10.3390/ma13194463

**Published:** 2020-10-08

**Authors:** Ahmed E. Abouelregal, Ibrahim-Elkhalil Ahmed, Mohamed E. Nasr, Khalil M. Khalil, Adam Zakria, Fawzy A. Mohammed

**Affiliations:** 1 Department of Mathematics, College of Science and Arts, Jouf University, Al-Qurayyat 77423, Saudi Arabia; i_elkhalil33@yahoo.com (I.-E.A.); mohamed.naser@fsc.bu.edu.eg (M.E.N.); Khalil.khalil@fsc.bu.edu.eg (K.M.K.); adammath2020@gmail.com (A.Z.); fawzy.noureldeen@sci.svu.edu.eg (F.A.M.); 2 Department of Mathematics, Faculty of Science, Mansoura University, Mansoura 35516, Egypt; 3 Department of Mathematics, Faculty of Science and Technology, Shendi University, Shendi 11111, Sudan; 4 Department of Mathematics, Faculty of Science, Benha University, P.O. 13518 Benha, Egypt; 5 Department of Mathematics, Faculty of Science, University of Kordofan, P.O. Box 160, North Kordofan, El-Obeid, Sudan; 6 Department of Mathematics, South Valley University, 83523 Qena, Egypt

**Keywords:** Moore–Gibson–Thompson heat equation, thermoelasticity, heat source, unbounded solid

## Abstract

Many attempts have been made to investigate the classical heat transfer of Fourier, and a number of improvements have been implemented. In this work, we consider a novel thermoelasticity model based on the Moore–Gibson–Thompson equation in cases where some of these models fail to be positive. This thermomechanical model has been constructed in combination with a hyperbolic partial differential equation for the variation of the displacement field and a parabolic differential equation for the temperature increment. The presented model is applied to investigate the wave propagation in an isotropic and infinite body subjected to a continuous thermal line source. To solve this problem, together with Laplace and Hankel transform methods, the potential function approach has been used. Laplace and Hankel inverse transformations are used to find solutions to different physical fields in the space–time domain. The problem is validated by calculating the numerical calculations of the physical fields for a given material. The numerical and theoretical results of other thermoelastic models have been compared with those described previously.

## 1. Introduction

The interaction of energy and matter is a complex area of study that has an impact on all aspects of everyday life, nature, and engineering. Energy may take various forms, including, but not limited to, thermal, kinetic, voltage, and chemical. Thermal energy, especially for mechanical engineers, is one of the most important energy forms. Solar thermal energy powers our meteorological processes and is a part of almost all kinds of innovations. When the function is not removed adequately, thermal energy may be dangerous, and hence, the issue of its transfer is actively studied. Conduction is the main form of heat conveyance. Pure conduction (diffusion) leads to a medium free of movement. In this case, microscopic particle collisions due to a temperature gradient move energy from more energy-intensive particles to less energy-intensive particles [1,2].

The internal energy of a thermodynamic system determines the energy inside of the system, which excludes the kinetic power of movement of the whole system and the potential energy of the whole system due to external force fields. It takes account of the system’s energy gains and losses due to internal changes. The inner energy is measured as a deviation from a normal status reference nil. The difference is determined by thermodynamic processes that take the system from the benchmark state to the current level of interest. The internal energy is one of the two cardinal status functions of the large variables of the state. Its value depends only on the current state of the system and does not depend on how many processes can be chosen between them. This is a thermodynamic potential that is connected to every thermodynamic potential. Microscopically, the internal energy can be measured by the kinetic strength of the translations, rotations, and movements of the microscopic motion particles of the device and by the potential energy of the microscopic force, including chemical bonds.

The branch of science that deals with the connection between heat and other forms such as work are described in thermodynamics. It is often summed up as three laws that define limitations on the interconvertibility of different forms of energy. The first law of thermodynamics states that internal energy rises equally to the total heat added plus environmental work. The first thermodynamics principle applies: it stays constant when it is an independent device. For an elastic medium, the mechanical energy term of the internal energy is expressed in terms of the stress and strain involved in the elastic processes. Elastic energy is the mechanical energy potential contained in a material or physical system structure, since it undergoes elastic deformation by work done thereon. When items are squeezed, scattered, or normally deformed in some way, elastic energy exists.

The thermoelastic foundations of elastic solids are based on the general laws of conventional thermodynamics, taking account of the energy conservation principle. The six components of the stress tensor and two-state characteristics of the temperature of the elastic body represent a complete set of state variables that define the system’s status. When at each point on the elastic spectrum these two state properties are defined, the system is defined completely, and the other state variables are known. We note that when a given state variable is represented as a unique function of a series of other state variables, this functional relationship is called the state equation.

The general term extended thermodynamics refers to many thermodynamic theories with three common features: (1) integrating fluxes in the formalism as independent variables, (2) extensive entropy and extendable entropy flux in conjunction with classical thermodynamic and the fluxes described, and (3) finding general transport equations for rapid and acute phenomena, which is consistent with the second law formulated on the basis of extended entropy and extended entropy flow. The motives of theory are particularly clear if the third point that was referred to starts: in a period of interest in miniaturizing devices and rapid operations, the practical need to form generalized transport equations for small systems and for rapid processes is particularly impressive. The computers may be a good example: based on the use of miniature, fast, dissipating heat, hence the use of hot, fast, and miniaturizing devices for conceptual thermodynamic reasons to discover the limits of the thermodynamics they can achieve [3].

The various developments of extended thermodynamics may vary from the three basic ideas referred in the above paragraph: (a) the given concrete fluxes; (b) the techniques employed in the exploitation of the second law; (c) the associations with the descriptive microscopy level; and (d) the number of systems under analysis.

In the analysis of unbalanced systems, the transport equations (that describe matter, along with thermodynamic equations) are crucial. In general, these flows are represented by gradients of a given physical sum (for example, the thermodynamic coupling of conserved variables). Indeed, heat flow, mass flux, and electric flow are expressed as gradients of the temperature, the chemical potentials, and electrical potentials, as thermodynamic forces (with forces known not as mechanical forces, but in a more general sense), in classical transport equations (Fourier law, Seebeck law, Ohm law, Soret law, Peltier law, Dufour law, etc.). In fact, a number of different couplings can depend on several powers [4].

Rationally expanded thermodynamics solve some problems that occur in ordinary thermodynamics. Initially inspired by the desire to overcome a heat-conduct paradox—a very esoteric mystery—the extended thermodynamics have developed into a predictive theory of light scattering. It now coincides with the kinetic theory of gases and has similar relationships with the hyperbolic framework mathematical theory [5]. Extended thermodynamics cover the numerous second sound phenomena, including heat diffusion, the transmission and accumulation of shear stress, and the second vibration in helium fluid, and semiconductors at low temperature. All these implications are normal for the symmetrical hyperbolic existence of field equations [5].

The above classical transport laws are of broad and useful validity, but they can be dismantled if disruption is too rapid or too steep. In fact, these calculations, from a microscopic point of view, require large collisions between the two opposite systems borders between heat carriers. The average time between consecutive particle collisions is called the average time of collision, and the aperture between two successive particles is called the average time of collision. If the device gets extremely uncommon or too small or if external disruptions are too rapid or too steep, the number of particle collisions during the test period is small, and the classical equations are not true anymore.

The interaction of high energy particles with high temperatures ensures that power is often transferred from hot to cold. Heat transfer via conduction is usually defined in Fourier’s law. The theory of thermoelasticity is a combination of heat conduction theory and elasticity theory. The heat causes the deformation of an elastic solid and the reciprocal effect of the deformation on the thermal state of the solid. It is known that when exposed to changes in temperature, most materials suffer from volumetric variations. The stresses resulting from these variations are referred to as thermal stresses. The classical theories of thermal elasticity are based on Fourier’s law of thermal conductivity, which in combination with other fundamental field equations leads to a mixture of deterministic fundamental equations. The thermal effects, away from the external mechanical convection, must be felt quickly through those equations. Hence, this principle enables unlimited rates of thermal destruction propagation. In problems involving very short timescales or high heat flow rates, this phenomenon is particularly pronounced.

Biot [6] proposed the principle of coupled thermoelasticity in order to eradicate the classic uncoupled principle’s inherent paradox. This paradox suggests that elastic changes have no temperature influence. The heat equations for both diffusion theories indicate that the heat wave propagation rates are unlike physical observations. Generalized thermoelasticity theories are designed to solve the weaknesses and shortcomings inherent in the classic dynamic thermoelasticity coupled theory, which enables the thermal signal to propagate with unlimited speed.

The first two popular generalized theories of thermoelasticity are the generalized models suggested by Lord and Shulman [7] and Green and Lindsay [8]. In the Lord and Shulman model [7], Fourier’s law is replaced by that of Maxwell–Cattaneo, which applies the heat conduction law to the thermal relaxation time, while in the second model [4], two relaxation times are incorporated into the constitutive relationships of stress tensors and entropy. Green and Naghdi propose the next generalization of thermoelasticity [9,10,11]. Their model is classified into three categories, which are regarded as thermoelasticity of types I, II, and III. Chandrasekharaiah [12] proposed the dual-phase-lag thermoelasticity model. This model relies in particular on the dual phase heat conduction model proposed by Tzou [13].

The dual phase heat conduction law may be a more generalized law involving two separate phase lags, one for the heat flow vector and the second for the temperature gradient, considering the effects of the microstructure in the heat transfer mechanism in order to catch the delayed reaction caused by the microstructure effects over time. Thermoelasticity with three-phase delays proposed by Roychoudhari is one of the most recent developments in the theory of thermoelasticity [14]. In addition to the phase lags of the heat flux vector and the temperature gradient, this model also features a phase delay of the thermal displacement gradient. These two proposals involve different derivatives when the heat flow and the temperature gradients are approached by the Taylor series, and one considers that Roychoudhari’s suggestion attempts to recover Green and Naghdi models if different Taylor approaches are taken into account. This new approach leads to various equations (according to the Taylor polynomial selected) to explain the heat conduction [15]. Some attempts have been made recently to modify the classical Fourier law using the time-derivative of a higher-order by Abouelregal [16,17,18].

Unfortunately, the two models (Tzou and Choudhuri) are leading Hadamard’s case into subjective problems. It has been proven that combining the modified Fourier law with the energy equation leads to a series of elements in the continuum of points such that the real component is infinitely dependent and thus the continuous dependency of solutions fails [16]. In recent years, the Moore–Gibson–Thompson (MGT) equation has gained great interest, and several articles aim to research and understand it [19,20,21,22,23]. This theory has been derived from a third-order differential equation, which has been incorporated into the meaning of some considerations of fluid dynamics [24]. A new thermoelastic model of heat conduction presented by the Moore–Gibson–Thompson equation has been constructed by Quintanilla [25]. The modified heat equation proposed by Quintanilla [25] has been achieved after applying the relaxation parameter in the Green–Naghdi Type III model. The number of papers devoted to this theory has grown significantly since the advent of Moore–Gibson–Thompson theory [26,27,28,29,30,31,32,33].

The purpose of this paper is to analyze the thermoelastic relationship of a continuous heat source with an infinite isotropic solid. The thermoelastic theory in which the heat conduction is estimated by the Moore–Gibson–Thompson equation has been presented. To solve the problem, we have used the potential function approach together with Laplace and Hankel technologies techniques to provide solutions in the transformed domain. Laplace transform inversion has been found numerically by assuming that the Laplace transform parameter is too high for the analytical temperature, displacement, and stress solutions that can be found in the time space domain. Numerical computations and graphic plots of the physical fields for copper material demonstrate the analytical solutions.

## 2. Basic Equations

Depending on the generalized thermoelasticity, in the absence of body forces, the field equations are considered for a linear, isotropic, and homogeneous solid that occupies the entire space.

The constitutive equations can be expressed as

(1) $$\sigma =\lambda \left(\mathrm{div}u\right)I+\mu \left[\nabla u+{\left(\nabla u\right)}^{Tr}\right]-\gamma \;\theta \;I,$$

where λ and μ are Lamé coefficients, $\theta =T-T_{0}$ denotes the increment of temperature with respect to the natural state $T_{0}$, u is the displacement vector, $\gamma =\left(3\lambda +2\mu \right)\alpha_{t}$, $\alpha_{t}$ is the coefficient of thermal expansion, σ is the stress tensor, I is the identity tensor, and suffix Tr is the transpose of the given vector. The relation between the strain tensor ε and displacements u is given by

(2) $$\varepsilon =\frac{1}{2}\left[\nabla u+{\left(\nabla u\right)}^{Tr}\right].$$

The equations of motion can be expressed as

(3) $$\mu \;\nabla^{2}u+\left(\lambda +\mu \right)\nabla \mathrm{div}u-\gamma \;\nabla \theta =\rho \;\ddot{u},$$

where ρ is the mass density.

Heat energy is transferred from one body to another only when the bodies have different temperatures. The basic concept in heat transfer is the classical Fourier law. The flow of heat q is related to the temperature gradient ∇θ according to the Fourier law by the following relation

(4) q=−K∇θ,

where K is the thermal conductivity.

The energy equation can be written as

(5) $$\rho C_{E}\frac{\partial \theta }{\partial t}+\gamma T_{0}\frac{\partial }{\partial t}\left(\mathrm{div}\left(u\right)\right)=-\mathrm{div}\left(q\right)+Q,$$

where $C_{E}$ denotes the specific heat at constant strain. The law of Fourier (1) with the energy Equation (5) provides a parabolic heat conduction equation that enables the propagation of waves at infinite speeds. The most popular theory is that of Maxwell and Cattaneo, which modifies the Fourier law by means of a constitutive equation containing a parameter of relaxation time as follows:

(6) $$\left(1+\tau_{0}\frac{\partial }{\partial t}\right)q=-K\nabla \theta ,$$

where $\tau_{0}$ is the relaxation parameter, which is assumed to be positive.

Green and Naghdi [9,10,11] presented another heat conduction model (GN-III), which is described by the constitutive equation

(7) $$q\left(x,t\right)=-\left[K\;\nabla \theta \left(x,t\right)+K^{*}\nabla \vartheta \left(x,t\right)\right],$$

where ϑ is the thermal displacement, $\dot{\vartheta }=\theta$, and $K^{*}$ is the thermal conductivity rate.

The model given in Equation (7) has the same shortcoming as the normal Fourier theory and predicts the instant propagation of heat waves. However, the principle of causality was not followed. So, it is also normal to modify this suggestion and include the constitutive equation of a relaxation factor to solve this problem [25]. Then, the modified heat conduction equation will be in the following form [25].

(8) $$\left(1+\tau_{0}\;\frac{\partial }{\partial t}\right)q\left(x,t\right)=-\left[K\;\nabla \theta \left(x,t\right)+K^{*}\nabla \vartheta \left(x,t\right)\right].$$

When Equations (6) to (8) are joined, we get the linear version of the heat conduction equation for isotropic material that is based on generalized Moore–Gibson–Thompson thermoelasticity (MGTE) [25,28]. The theory of MGTE is the generalization of the theory of Lord–Shulman (LS) [7] and of type III Green-Naghdi theory of thermoelasticity (GN-III) [9,10].

(9) $$\left(1+\tau_{0}\;\frac{\partial }{\partial t}\right)\left[\rho C_{E}\frac{\partial^{2}\theta }{\partial t^{2}}+\gamma T_{0}\frac{\partial^{2}}{\partial t^{2}}\left(\mathrm{div}\left(u\right)\right)-\frac{\partial Q}{\partial t}\right]=K\;\nabla^{2}\dot{\theta }+K^{*}\nabla^{2}\theta .$$

### Special Cases

The Moore–Gibson–Thompson (MGTE) thermoelasticity model is limited to four separate models of generalized thermoelasticity in some special cases. The cases that have been earned are classified as:

- The classical thermoelastic model (CTE) is possible when $\tau_{0}=K^{*}=0$.
- Lord and Shulman’s model (LS) can be acquired as a limited case when $K^{*}=0$.
- The introduced model makes it possible to obtain Green and Naghdi theory (GN-II) when $\tau_{0}=K=0$.
- The theory of Green and Naghdi of type III (GN-III) can be obtained if $\tau_{0}=0$.

## 3. Formulation of the Problem

In this article, we assume a continuous line heat source in an infinite body located along the $x_{3}$-axis. We use the spherical polar coordinates $\left(r,\Theta ,\psi \right)$ of origin in the center of space and take into account the spherically symmetric thermal interactions. Thus, the thermoelastic interactions in the medium are axisymmetric in nature, which denote the displacement and the temperature as $u=u\left(r,t\right)$ and $\theta =\theta \left(r,t\right)$ in which the distance r is calculated from the axis. This means that the stress tensor has only two components in the radial and transverse directions ($\sigma_{rr}$ and $\sigma_{\Theta \Theta }$).

The regularity conditions are taken in the light of the considered problem as

(10) $$u\left(r,t\right),\;\;\theta \left(r,t\right),\;\sigma_{rr}\left(r,t\right),\sigma_{\Theta \Theta }\left(r,t\right)\to 0\;\;\;\mathrm{as}\;\;r\to \infty .$$

Initially, we assume that the body has no deformation, has the reference temperature $T_{0}$, and also a zero rate of temperature change. Therefore, initial conditions may be expressed as

(11) $$u\left(r,0\right)=\frac{\partial u\left(r,0\right)}{\partial r}=0,\;\;\;\theta \left(r,0\right)=\frac{\partial \theta \left(r,0\right)}{\partial r}=0.$$

The component of the strain tensor is given by

(12) $$e_{rr}=\frac{\partial u}{\partial r},\;\;e_{\Theta \Theta }=\frac{u}{r}\;=e_{\phi \phi },e_{r\Theta }=e_{r\phi }=e_{\Theta \phi }=0.$$

The Duhamel–Neumann relations are reduced to

(13) $$\sigma_{rr}=2\mu \frac{\partial u}{\partial r}+\lambda e-\gamma \theta ,$$

(14) $$\sigma_{\Theta \Theta }=2\mu \frac{u}{r}+\lambda e-\gamma \theta =\sigma_{\psi \psi }.$$

In the spherical coordination, the dynamic equation without body forces can be written as

(15) $$\frac{\partial \sigma_{rr}}{\partial r}+\frac{2}{r}\left(\sigma_{rr}-\sigma_{\Theta \Theta }\right)=\rho \frac{\partial^{2}u}{\partial t^{2}}.$$

The equation of motion (15) will be presented by using Equations (13) and (14).

(16) $$\left(\lambda +2\mu \right)\left[\frac{\partial^{2}u}{\partial r^{2}}+\frac{2}{r}\frac{\partial u}{\partial r}-\frac{2u^{2}}{r^{2}}\right]-\gamma \frac{\partial \theta }{\partial r}=\rho \frac{\partial^{2}u}{\partial t^{2}}.$$

The thermoelastic model of Moore–Gibson–Thompson (MGT) (9) can be expressed as

(17) $$\left(1+\tau_{0}\;\frac{\partial }{\partial t}\right)\left[\rho C_{E}\frac{\partial^{2}\theta }{\partial t^{2}}+\gamma T_{0}\frac{\partial^{2}}{\partial t^{2}}\left(\frac{\partial u}{\partial r}+\frac{2u}{r}\right)-\frac{\partial Q}{\partial t}\right]=\;\left[K\frac{\partial }{\partial t}+K^{*}\right]\nabla^{2}\theta ,$$

where the operator of the Laplacian $\nabla^{2}$ is given by

(18) $$\nabla^{2}\theta =\frac{\partial^{2}\theta }{\partial r^{2}}+\frac{2}{r}\frac{\partial \theta }{\partial r}.$$

## 4. Solution of the Problem

We now present the following non-dimensional variables to solve the problem

(19) $$\begin{array}{cccc} r^{\prime }=\upsilon \eta r, & u^{\prime }=\upsilon \eta u, & t^{\prime }=\upsilon^{2}\eta t, & \theta ’=\frac{\theta }{T_{0}},\\ & \sigma {’}_{ij}=\frac{\sigma_{ij}}{\lambda +2\mu }, & Q^{\prime }=\frac{\rho \gamma Q}{\rho K\upsilon^{4}\eta^{2}}. & \end{array}$$

where $\eta =\rho C_{E}/K$ and $\upsilon =\sqrt{\left(\lambda +2\mu \right)/\rho }$ denote the velocity of the longitudinal wave. The governing Equations (13)–(17) are reduced to non-dimensional forms with the help of Equation (19) as

(20) $$\sigma_{rr}=\frac{\partial u}{\partial r}+\lambda_{1}\frac{u}{r}-b_{1}\theta ,$$

(21) $$\sigma_{\Theta \Theta }=\lambda_{1}\frac{\partial u}{\partial r}+\frac{u}{r}-b_{1}\theta =\sigma_{\psi \psi },$$

(22) $$\frac{\partial^{2}u}{\partial r^{2}}+\frac{2}{r}\frac{\partial u}{\partial r}-\frac{2u^{2}}{r^{2}}-\varepsilon_{2}\frac{\partial \theta }{\partial r}=\frac{\partial^{2}u}{\partial t^{2}},$$

(23) $$\left(1+\tau_{0}\;\frac{\partial }{\partial t}\right)\left[\frac{\partial^{2}\theta }{\partial t^{2}}+b_{2}\frac{\partial^{2}}{\partial t^{2}}\left(\frac{\partial u}{\partial r}+\frac{2u}{r}\right)-\frac{1}{b_{1}}\frac{\partial Q}{\partial t}\right]=\;\left(a_{0}+\frac{\partial }{\partial t}\right)\nabla^{2}\theta ,$$

where

(24) $$\lambda_{1}=\frac{\lambda }{\lambda +2\mu },\;\;\;b_{1}=\frac{\gamma T_{0}}{\lambda +2\mu }\;\;\;b_{2}=\frac{\gamma }{\rho C_{E}},\;a_{0}=\frac{K^{*}}{\upsilon^{2}\eta K}.$$

In the non-dimensional Equations (20)–(23), the primes are dropping for convenience.

The thermoelastic potential function φ can be defined as

(25) $$u=\frac{\partial \varphi }{\partial r}.$$

The function φ is introduced in Equations (20)–(23) to get

(26) $$\sigma_{rr}=\left(\lambda_{1}-1\right)\frac{1}{r}\frac{\partial \varphi }{\partial r}+\frac{\partial^{2}\varphi }{\partial t^{2}},$$

(27) $${\bar{\sigma }}_{\Theta \Theta }=\left(\lambda_{1}-1\right)\frac{\partial^{2}\varphi }{\partial r^{2}}+\frac{\partial^{2}\varphi }{\partial t^{2}},$$

(28) $$\left(1+\tau_{0}\;\frac{\partial }{\partial t}\right)\left[\frac{\partial^{2}\theta }{\partial t^{2}}+b_{2}\frac{\partial^{2}}{\partial t^{2}}\nabla^{2}\varphi -\frac{1}{b_{1}}\frac{\partial Q}{\partial t}\right]=\;\left(a_{0}+\frac{\partial }{\partial t}\right)\nabla^{2}\theta ,$$

(29) $$\nabla^{2}\varphi -b_{1}\theta =\frac{\partial^{2}\varphi }{\partial t^{2}}.$$

The removal of θ between Equations (28) and (29) gives

(30) $$.\begin{array}{c} \;\left(a_{0}+\frac{\partial }{\partial t}\right)\nabla^{4}\varphi -\frac{\partial^{2}}{\partial t^{2}}\left[a_{0}+\frac{\partial }{\partial t}+\left(1+\varepsilon \right)\left(1+\tau_{0}\;\frac{\partial }{\partial t}\right)\right]\nabla^{2}\varphi \\ +\frac{\partial^{4}}{\partial t^{4}}\left(1+\tau_{0}\;\frac{\partial }{\partial t}\right)\varphi =-\frac{1}{b_{1}}\left(1+\tau_{0}\;\frac{\partial }{\partial t}\right)\frac{\partial Q}{\partial t}. \end{array}.$$

We will consider the existence of a continuous heat source in the origin that can be expressed by [33]

(31) $$Q\left(r,t\right)=\frac{Q_{0}\delta \left(r\right)H\left(t\right)}{\left(2\pi r\right)},$$

where $Q_{0}$ is constant, $\delta \left(r\right)$ is the Dirac’s delta function, and $H\left(t\right)$ is the Heaviside unit step function.

## 5. Solution in the Transformed Domains

We use the Laplace transformation defined by the following relation for the solution of the problem

(32) $$\bar{f}\left(r,s\right)=\overset{\infty }{\underset{0}{\int }}f\left(r,t\right)e^{-st}dt.$$

Under the initial conditions and applying the Laplace transform to the basic equations, we get

(33) $${\bar{\sigma }}_{rr}=\left(\lambda_{1}-1\right)\frac{1}{r}\frac{d\bar{\varphi }}{dr}+s^{2}\bar{\varphi ,}$$

(34) $${\bar{\sigma }}_{\Theta \Theta }=\left(\lambda_{1}-1\right)\frac{d^{2}\bar{\varphi }}{dr^{2}}+s^{2}\bar{\varphi ,}$$

(35) $$b_{1}\bar{\theta }=\left(\nabla^{2}-s^{2}\right)\bar{\varphi },$$

(36) $$\begin{array}{c} \left(a_{0}+s\right)\nabla^{4}\bar{\varphi }-s^{2}\left[a_{0}+s+\left(1+\varepsilon \right)\left(1+\tau_{0}\;s\right)\right]\nabla^{2}\bar{\varphi ,}\\ +s^{4}\left(1+\tau_{0}\;s\right)\bar{\varphi }=-\frac{Q_{0}}{2\pi rb_{1}}\left(1+\tau_{0}\;s\right)\delta \left(r\right). \end{array}$$

Equation (36) can be rewritten as

(37) $$\left(\nabla^{2}-m_{1}^{2}\right)\left(\nabla^{2}-m_{2}^{2}\right)\bar{\varphi }=-\frac{\;\alpha }{r}\delta \left(r\right),$$

where $m_{i}^{2}$, $\left(i=1,2\right)$ are the roots of the equation

(38) $$m^{4}-Am^{2}+B=0,$$

where

(39) $$A=\frac{s^{2}\left[a_{0}+s+\left(1+\varepsilon \right)\left(1+\tau_{0}\;s\right)\right]}{\;\left(a_{0}+s\right)},B=\frac{s^{4}\left(1+\tau_{0}\;s\right)}{\;\left(a_{0}+s\right)},\alpha =\frac{Q_{0}\left(1+\tau_{0}\;s\right)}{2\pi b_{1}\left(a_{0}+s\right)}.$$

Now, we use the Hankel transformation described by

(40) $$\hat{f}\left(\xi ,s\right)=\overset{\infty }{\underset{0}{\int }}rJ_{0}\left(\xi r\right)\bar{f}\left(r,s\right)dr,$$

where $J_{0}\left(\xi r\right)$ refers to the first kind and the zero order function of Bessel. Using the Hankel transform, Equation (37) can be transformed into the following form

(41) $$\left(\xi^{2}+m_{1}^{2}\right)\left(\xi^{2}+m_{2}^{2}\right)\hat{\varphi }=-\alpha .$$

The inversion transformation of Hankel can be defined by

(42) $$\bar{f}\left(x,s\right)=\int_{0}^{\infty }\xi J_{0}\left(\xi r\right)\hat{f}\left(\xi ,s\right)d\xi .$$

Then, the solution of the function $\bar{\varphi }\left(r,s\right)$ can be obtained in the Laplace transform field as

(43) $$\bar{\varphi }\left(r,s\right)=\frac{\alpha }{\left(m_{1}^{2}-m_{2}^{2}\right)}\left[K_{0}\left(m_{1}r\right)-K_{0}\left(m_{2}r\right)\right],$$

where $K_{0}\left(m_{i}r\right)$ is the second kind of the modified Bessel function of zero order. Now, we will use the following recurrent relationship for the second kind of modified Bessel function $K_{n}\left(m_{i}r\right)$:

(44) $$\frac{d}{dz}\left(K_{0}\left(m_{i}r\right)\right)=-m_{i}K_{1}\left(m_{i}r\right),\;\nabla^{2}\left(K_{0}\left(m_{i}r\right)\right)=m_{i}^{2}\;K_{0}\left(m_{i}r\right),$$

where the function $K_{1}\left(k_{i}r\right)$ is the second kind of the modified Bessel function of order one.

Substituting Equation (43) into Equation (35), we get

(45) $$\bar{\theta }=\frac{\alpha }{b_{1}\left(m_{1}^{2}-m_{2}^{2}\right)}\left[\left(m_{1}^{2}-s^{2}\right)\;K_{0}\left(m_{1}r\right)-\left(m_{2}^{2}-s^{2}\right)K_{0}\left(m_{2}r\right)\right].$$

By inserting Equation (43) into (25) in the Laplace transformation, we get

(46) $$\bar{u}\left(r,s\right)=-\frac{\alpha }{\left(m_{1}^{2}-m_{2}^{2}\right)}\left[m_{1}K_{1}\left(m_{1}r\right)-m_{2}K_{1}\left(m_{2}r\right)\right].$$

Substituting Equation (43) in Equations (33) and (34), we obtain the thermal stresses ${\bar{\sigma }}_{rr}$ and ${\bar{\sigma }}_{\Theta \Theta }$ as

(47) $${\bar{\sigma }}_{rr}=\frac{\alpha s^{2}}{\left(m_{1}^{2}-m_{2}^{2}\right)}\left[K_{0}\left(m_{1}r\right)-K_{0}\left(m_{2}r\right)\right]-\frac{\alpha \left(\lambda_{1}-1\right)}{r\left(m_{1}^{2}-m_{2}^{2}\right)}\left[m_{1}K_{1}\left(m_{1}r\right)-m_{2}K_{1}\left(m_{2}r\right)\right],$$

(48) $$\begin{array}{c} {\bar{\sigma }}_{\Theta \Theta }=\frac{\alpha }{2\left(m_{1}^{2}-m_{2}^{2}\right)}[\left(2s^{2}+m_{1}^{2}\left(-1+\lambda_{1}\right)\right)K_{0}\left(m_{1}r\right)+\left(m_{2}^{2}-2s^{2}-m_{2}^{2}\lambda_{1}\right)K_{0}\left(m_{2}r\right)\\ \left(-1+\lambda 1\right)\left(m_{1}^{2}K_{2}\left(m_{1}r\right)-m_{2}^{2}K_{2}\left(m_{1}r\right)\right)] \end{array}.$$

The system of Equations (43)–(48) provides the solutions of temperature, stresses, and displacement in the Laplace transform domain. The solutions in the $\left(r,t\right)$ domain can be achieved by inverting the Laplace transformation.

A detailed and efficient numerical technique is used in this paper to obtain the Laplace transforms inversion. An extension of the Fourier series [34], a numerical inversion technique, has been applied. This approach approximates the inversion of the Laplace transform using the relation

(49) $$g\left(x,t\right)=\frac{e^{ct}}{t_{1}}\left(\frac{g\left(c\right)}{2}+Re\overset{N}{\underset{k=1}{\sum }}e^{ik\pi t/t_{1}}g\left(c+ik\pi /t_{1}\right)\right),\;\;0\leq t\leq 2t_{1}$$

where N is the large enough integer of the truncated infinite Fourier series representing a number of terms. The parameter N must be chosen as follows

(50) $$e^{ct}Re\left(e^{iN\pi t/t_{1}}g\left(c+iN\pi /t_{1}\right)\right)\leq \varepsilon_{1},$$

where $\varepsilon_{1}$ is a small positive number persecuted that corresponds to the degree of precision to be achieved. The coefficient c is a free positive parameter, which must be larger than the real parts of all singularities of $\bar{g}\left(x,t\right)$. The parameter c was optimized in accordance with the requirements mentioned in [34].

## 6. Results and Discussion

To explain the previously mentioned analytical method, we are now considering a numerical illustration to provide computational results. These results show changes in temperature and displacement as well as radial stress and hoop stress. The problem has been solved numerically for the special case of a copper material. The following physical constant values are taken for this reason as [35]:

$$\lambda =7.76\times \frac{10^{10}N}{m^{2}},\;\mu =3.86\times \frac{10^{10}N}{m^{2}},\;\alpha_{t}=1.78\times 10^{-5}K^{-1},T_{0}=1K,$$

$$K=386{\mathrm{Wm}}^{-1}K^{-1},\;\;C_{E}=383.1\;J/\left(\mathrm{Kkg}\right),\;Q_{0}{=1\mathrm{Wm}}^{-2},\;\rho =8.954\times 10^{3}\mathrm{kg}/m^{3}.$$

**Figure 1:**
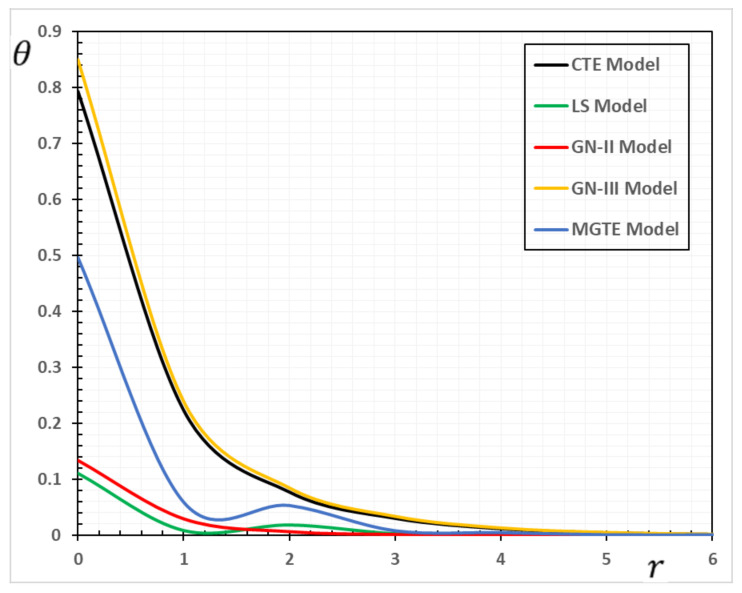
The temperature variation θ for different models of thermoelasticity when the time instant t=0.12.

**Figure 2:**
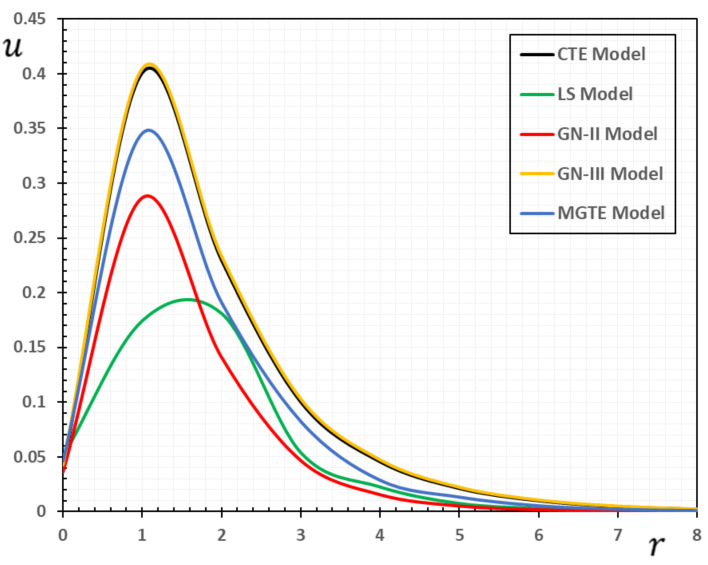
The displacement variation u for different models of thermoelasticity when the time instant t=0.12.

**Figure 3:**
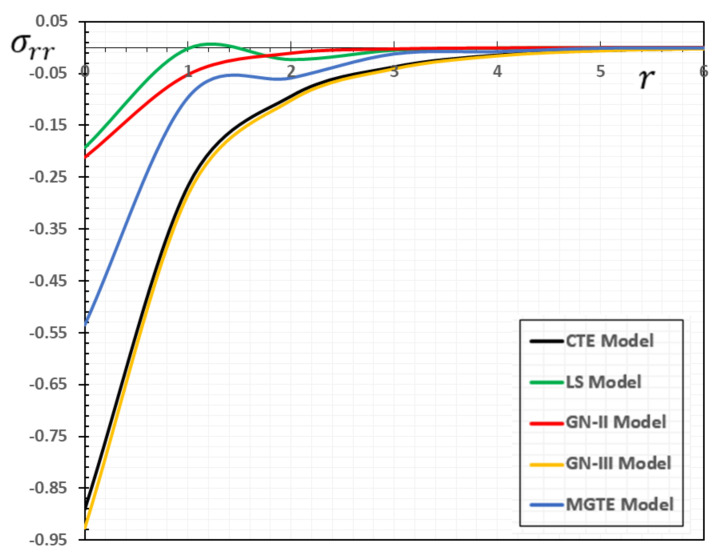
The stress variation $\sigma_{rr}$ for different models of thermoelasticity when the time instant t=0.12.

**Figure 4:**
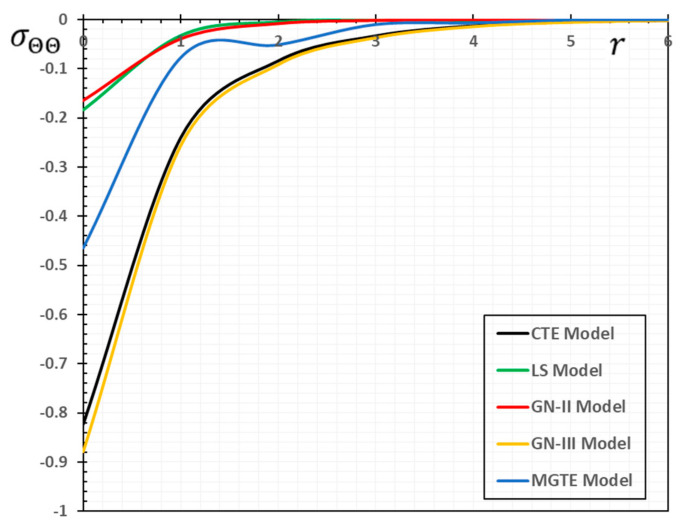
The stress variation $\sigma_{\Theta \Theta }$ for different models of thermoelasticity when the time instant t=0.12.

**Figure 5:**
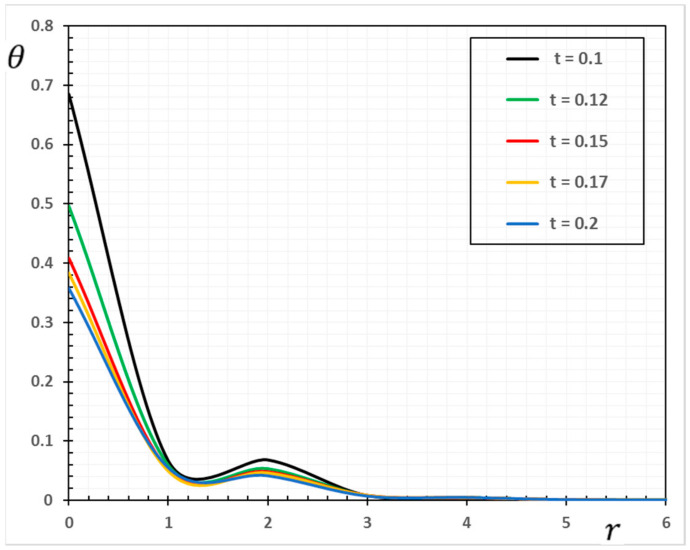
The variation of the temperature θ against radial distance r and time t.

**Figure 6:**
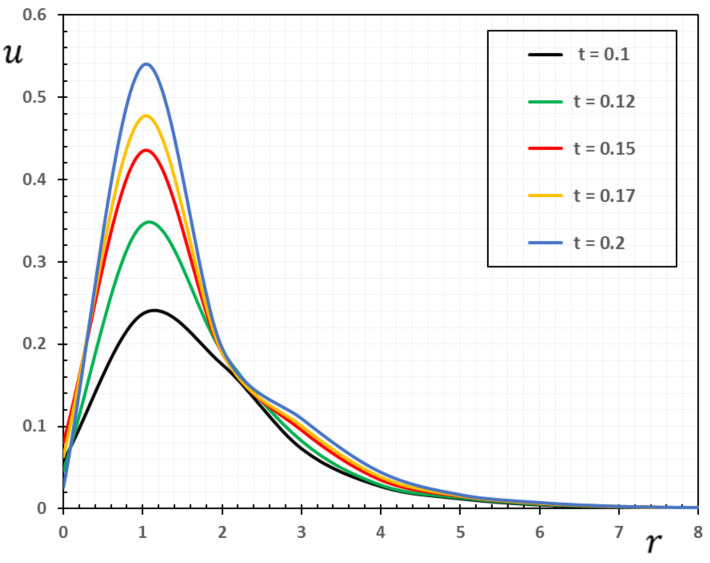
The variation of the displacement u against radial distance r and time t.

**Figure 7:**
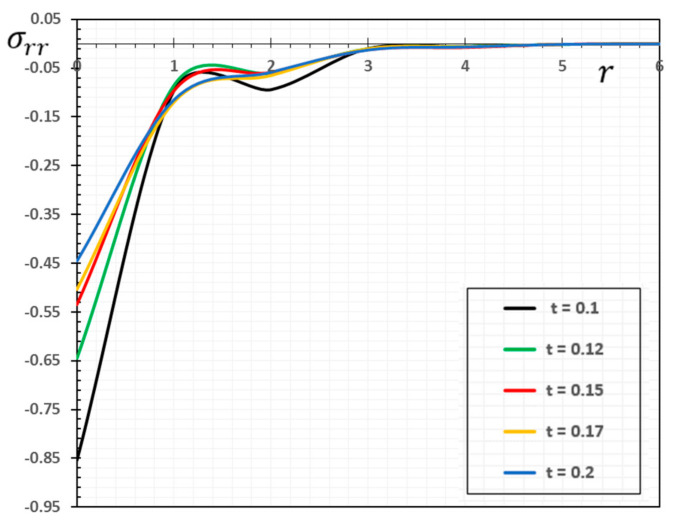
The variation of the stress $\sigma_{rr}$ against radial distance r and time t.

**Figure 8:**
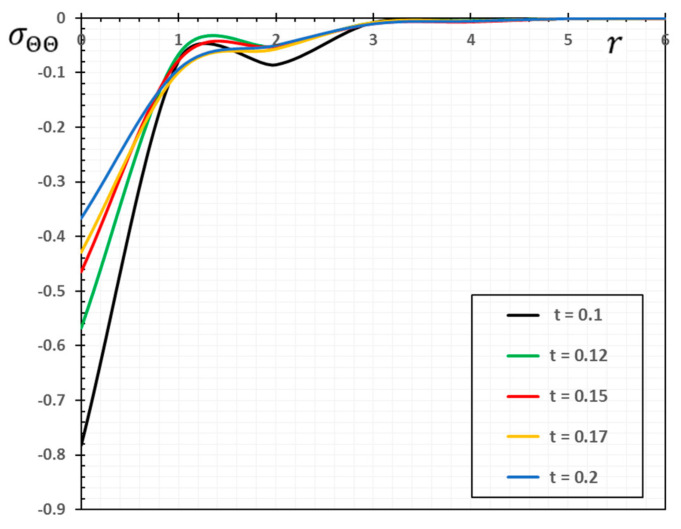
The variation of the stress $\sigma_{\Theta \Theta }$ against radial distance r and time t.

In the sense of the generalized Moore–Gibson–Thompson thermoelasticity (MGTE) [25,29], dimensionless numerical results are analyzed for the distributions of temperature θ, radial displacement u, as well as the thermal stresses $\sigma_{rr}$ and $\sigma_{\Theta \Theta }$ inside the body. Table 1, Table 2, Table 3 and Table 4 and Figure 1, Figure 2, Figure 3, Figure 4, Figure 5, Figure 6, Figure 7 and Figure 8 show the effects of the physical fields in the radial direction of the solid. The figures were divided into two groups: the first includes graphs that differ only with distance for the fields studied, and the second includes three-dimensional figures with changes in time and distance. This section includes some numerical findings in tables that can be used for future researchers’ comparisons, comparisons between different thermoelasticity models, and for practical purposes. The variances of the field variables versus the distance r for the CTE, LS, GN-II, GN-III models and MGTE model when the time t=0.12 are displayed in Table 1, Table 2, Table 3 and Table 4. 

The temperature variation θ along the radial distance r is shown in Table 1. It is evident from Figure 1 that temperature has a non-zero value only in a finite space area at a given time. The disturbance outside this area disappears, which means that the area has not yet felt the thermal turbulence. At various points, the non-zero region transports over time correspondingly. This means that the heat transports through the medium as a wave with finite speed. This is very different from conventional thermoelastic models, in which the infinite diffusion velocity is intrinsic.

The maximal values of the temperature θ are measured when r=0 after which the temperature decreases slowly to zero with the rise to distance r. From Figure 1, it shows the difference between the predictions of GN-III and MGTE theories. It is also shown that the magnitude of θ for the GN-III model is larger than that of MGTE, while the graphs in the LS and MGTE models reflect similar results for both models.

The results of the generalized GN-III thermoelasticity model are evident from the tables in that they differ significantly from those of the GN-II thermoelasticity model with few values for energy dissipation. Moreover, the presence of the relaxation parameter in both LS and MGTE models could show a slowing of the temperature decay.

In the extended thermoelasticity theories proposed by Sherief and Anwar [35], Chandrasekharaiah and Murthy [36], and Ezzat [37], very similar behavior of the temperature spectrum behind and beyond the elastic wave front should be noted here. However, the different temperature field behaviors of the thermoelasticity GN-II type were stated by Prasad et al. [36], Dhaliwal et al. [39], and Chandrasekharaiah and Srinath [40].

Prasad et al. [38] showed that under all these theories, there is a discontinuity of the temperature field with the constant jumping on the two wave fronts, but with radial distance in MGTE’s thermoelasticity model, the temperature field is continuously decreasing. 

Table 2 shows the change in displacement u over the distance r. Obviously, the displacement begins with the minimum values when r=0; then, it gradually increases to the peak values and then decreases to zero. It is also observed that for the MGTE theory, the decay of the displacement u is faster than that of the CTE theory. In addition, it is observed that the magnitude of the displacement u for GN-III theory is larger than that of MGTE theory. Moreover, it was found that the displacement values of the GN-III and CTE theories were greater than those of the GN-II and LS models in addition to the MGTE model.

From Figure 2, it can be seen that the inner points of the medium undergo expansion deformation due to the presence of the thermal source $Q\left(r,t\right)$. In other words, we can say that deformation is a dynamic process. The table and figure indicate that the displacement distribution travels to the depth of the medium at a finite speed over time. With the passage of time, the turbulence zone gradually moves within the medium, becomes larger and larger near the source, and then gradually decreases again until it fades to zero. Over time, the expansion area is steadily moving within and rising bigger and larger. This raises the radial displacement of the cylinder surface. At a certain moment, the radial displacement non-zero area is finite, owing to the wave effect of heat. It shows that heat moves to the depth of the medium with a finite speed over time. The faster the heat moves, the greater the thermal disturbance and the radial displacement.

Table 3 demonstrates the variations in the stress $\sigma_{rr}$ versus radial distance r. It is observed that the maximum negative value is reached after it is gradually increased to zero. The $\sigma_{rr}$ magnitude is also shown to be larger for the GN-III model compared to all other models. From the figure and the table, it can be seen that the presence of the parameter of relaxation is likely to reduce the stress $\sigma_{rr}$ profile. The fact that the waves propagate at finite velocities is illustrated in the generalized thermal elasticity in the case of the MGTE model, as shown in Figure 3 and Table 3. The tensile stress region can also be seen expanding, while the compressed area becomes smaller over time, which is consistent with the above-mentioned dynamic stretching effect. In addition to the above, we notice that tensile stress is endured by the medium near the heat source. This is consistent with the deformation of the radial expansion medium. The tensile stress region can also be found to become bigger as the compressed area becomes smaller with the time that corresponds to the above-mentioned dynamic expansion effect.

For CTE or the GN-II, I as stated in [35,36] and [37], the pattern of variability in radial stress outside thermoelastic obverse is also very similar in this case. However, this region has a different pattern according to LS, MGTE, and GN-II models, noting that the radial stress is a tensile only behind the elastic wave front [39,40], and the absolute value of the radial stress increases beyond the thermoelastic wave fronts.

Table 4 and Figure 4 are presented to study the variation in the amount of hoop stress $\sigma_{\Theta \Theta }$ versus x for different thermoelastic models. It is evident from the numerical values and figures that the mechanical waves of the stress field $\sigma_{\Theta \Theta }$ propagate at finite speeds within the medium in the case of the presented theory of thermoelasticity (MGTE).

The difference between the MGTE model and the other models of thermoelasticity when t=0.12 is provided in Table 4 and Figure 4. In comparison, with the change in distance x for all models, the magnitude of $\sigma_{\Theta \Theta }$ increases. In the variation of the stress $\sigma_{\Theta \Theta }$, a similar phenomenon for the stress $\sigma_{\Theta \Theta }$ can also be seen in Table 4 and Figure 4. From the numerical values and the figure, we find that the MGTE theory is more realistic than CTE and GN-III. In contrast with circumferential stress $\sigma_{\Theta \Theta }$ in each case, the radial stress $\sigma_{\mathrm{rr}}$ starts with a high negative value. In the current context, the radial, as well as the circumferential stresses on the thermal wave front, show proximal actions in the same way as in the GN-II model (see [39,40]), so this is more significant at the time for smaller values. 

Numerical results of field quantities from the MGTE model are shown as 2D graphs compared to the distance r for different values of time instant t (t=0.12, 0.15, 0.17 and 0.20) in Figure 5, Figure 6, Figure 7 and Figure 8. It has been observed through the graphs that some values of the related quantities differ with the values of the parameters r and t. From the presented figures, it is clear that time plays an important role in all the studied physical distributions. It must be concluded that the higher absolute value of these profiles for all distributions except for the temperature θ occurs with increasing time. The area of influence is directly proportional to time, which means that with any change in time, the area of influence increases.

The temperature variation θ shown in Figure 5 has a maximum value at r=0 for all time periods and decreases with increasing radial distance r. In addition, Figure 6 shows that higher time values cause an increase in the displacement values. Moreover, as shown in Figure 7 and Figure 8, it has been found that the values of thermal stresses $\sigma_{rr}$ and $\sigma_{\Theta \Theta }$ near the heat source increase with increasing time and with increasing distance; we find that the stresses increase in some ranges and decrease in others with increasing parameters r and t until they diminish.

An important observation noted in the context problem, where the mediator is of an indefinite extent, is that the solution to any of the field variables studied for the MGTE model fades to indistinguishable outside a finite area of space. This clearly illustrates the contradiction between the generalized and coupled thermoelastic theories. In the classical coupled theory, the thermal waves propagate at rates of infinite velocity, so the estimate for any field variable is not zero (although it may be very small) for any large estimate of distance r. In the Moore–Gibson–Thompson (MGT) model proposed by Quintanilla [25], the reaction to thermal effects does not achieve a temporary expansion but rather remains in a limited region of space that stretches with the progression of time.

As in the CTE, LS, and GN-II theories, the heat source effects of the present state are limited to a finite region but are time-dependent from the space surrounding the heat source. This article demonstrates that the temperature, the hoop, and the axial stresses are particularly sensitive to certain locations and times at what is known as the temperature wave front. From the graphs, the estimated results are in line with those of the current literature [38].

## 7. Concluding Remarks

In the current study, the heat wave distribution is investigated by the application of the established generalized Moore–Gibson–Thompson thermoelastic model (MGTE) in a homogeneous isotropic linear thermoelastic solid. The numerical calculation was performed by taking a copper-like substance, and the theoretical predictions were explained with different tables and figures after obtaining the analytical results of the studied variables such as temperature, displacement, and radial and circumferential stresses in the domain of Laplace transform.

After a certain time, the points of the considered body that lie beyond the faster wave front see no deterioration. This phenomenon is a feature of all generalized models of thermoelasticity. Thus, our perceptions confirm that Quintanilla’s theory [25] is in fact a general theory of thermoelasticity. This observation is consistent with previous theoretical results. Although the tables and figures are self-explanatory in showing the various properties that occur in wave propagation, it is also evident from the results that the area of influence for each physical field is directly proportional to time, which means that the area of influence increases with increasing time.

The results can extend to include a variety of geophysical problems associated with temperature change. Physical implementation includes issues such as field fires, gas operations, etc. This problem is also useful in geo-mechanics, where attention is focused on the many marvels of seismic tremors and the computation of temperature, displacement, and stress distributions due to different sources.

## Figures and Tables

**Table 1:** The temperature variation θ against the radial distance r.

r	CTE	LS	GN-II	GN-III	MGTE
0	0.793718	0.467859	0.133866	0.830122	0.496417
1	0.223291	0.052589	0.0294219	0.239111	0.0591109
2	0.0772751	0.0487031	0.00643665	0.0846638	0.0532864
3	0.030374	0.00702464	0.0014042	0.0337784	0.00811275
4	0.0116987	0.00413792	0.000315335	0.0132107	0.00470505
5	0.00442973	0.000721301	7.15311 × 10^−5^	0.00509094	0.000870387
6	0.00169297	0.000653428	1.63496 × 10^−5^	0.0019812	0.000752963
7	0.000655808	8.31318 × 10^−5^	3.76 × 10^−6^	0.000781042	0.000103645
8	0.000255796	1.82117 × 10^−5^	8.71 × 10^−7^	0.000309935	2.38765 × 10^−5^
9	0.000100076	9.01 × 10^−6^	2.03 × 10^−7^	0.000123373	1.16608 × 10^−5^
10	3.92668 × 10^−5^	2.32 × 10^−6^	4.73 × 10^−8^	0.000049258	3.11 × 10^−6^

**Table 2:** The displacement variation u against the radial distance r.

r	CTE	LS	GN-II	GN-III	MGTE
0	0.0394373	0.050189	0.036187	0.0396386	0.0463166
1	0.400918	0.174288	0.286732	0.404637	0.345537
2	0.229875	0.181084	0.141088	0.233535	0.19115
3	0.100204	0.0539699	0.0466778	0.102894	0.0823943
4	0.0456167	0.0225611	0.0155246	0.0473761	0.0286583
5	0.0212324	0.00750074	0.0051575	0.0223083	0.013251
6	0.00997956	0.0029345	0.00173138	0.0106099	0.00540503
7	0.00469392	0.001027	0.000563854	0.00505338	0.00218025
8	0.00222833	0.000342003	0.000184689	0.00242982	0.000945051
9	0.00106746	0.000139875	0.0000608047	0.00117907	0.000365592
10	0.000515338	0.0000458229	0.0000201294	0.000576615	0.000165771

**Table 3:** The stress variation $\sigma_{rr}$ against the radial distance r.

r	CTE	LS	GN-II	GN-III	MGTE
0	−0.890415	−0.192783	−0.211612	−0.926499	−0.534644
1	−0.267014	−0.00184061	−0.0520104	−0.283606	−0.0961256
2	−0.0937151	−0.0230486	−0.0107591	−0.101745	−0.0586502
3	−0.0371652	−0.00500324	−0.00263878	−0.0409447	−0.0122027
4	−0.0143192	−0.000965933	−0.000605306	−0.016025	−0.00780437
5	−0.00544072	−0.000126451	−0.000136567	−0.00619642	−0.0012295
6	−0.0020871	−0.000109581	−0.0000318376	−0.00241978	−0.000713513
7	−0.000809845	−0.000026613	−7.36742 × 10^−6^	−0.000955483	−0.000142457
8	−0.000316175	−5.04421 × 10^−6^	−1.70584 × 10^−6^	−0.000379521	−0.0000490001
9	−0.000123815	−1.22885 × 10^−6^	−3.98285 × 10^−7^	−0.000151214	−0.0000165664
10	−0.0000486253	−5.83347 × 10^−7^	−9.30972 × 10^−8^	−0.0000604276	−4.10155 × 10^−6^

**Table 4:** The stress variation $\sigma_{\Theta \Theta }$ against radial distance r.

r	CTE	LS	GN-II	GN-III	MGTE
0	−0.823	−0.183878	−0.164702	−0.858651	−0.464514
1	−0.240197	−0.0327232	−0.039354	−0.256174	−0.0774585
2	−0.0841031	−0.00508705	−0.00835588	−0.0917245	−0.0516142
3	−0.0333546	−0.000636734	−0.00197767	−0.0369129	−0.0102023
4	−0.0128787	−0.00158496	−0.00045194	−0.0144746	−0.00697777
5	−0.00489277	−0.000217583	−0.000102402	−0.00559616	−0.00102648
6	−0.0018758	−0.000040815	−0.0000237646	−0.00218427	−0.000635166
7	−0.000727943	−8.19272 × 10^−6^	−5.49842 × 10^−6^	−0.000862603	−0.000120468
8	−0.000284282	−1.65229 × 10^−6^	−1.27454 × 10^−6^	−0.000342719	−0.0000420921
9	−0.000111343	−2.74578 × 10^−7^	−2.97465 × 10^−7^	−0.00013657	−0.0000141679
10	−0.0000437302	−3.12309 × 10^−7^	−6.95505 × 10^−8^	−0.0000545793	−3.40854 × 10^−6^
